# Variability in school closure decisions in response to 2009 H1N1: a qualitative systems improvement analysis

**DOI:** 10.1186/1471-2458-11-73

**Published:** 2011-02-01

**Authors:** Tamar Klaiman, John D Kraemer, Michael A Stoto

**Affiliations:** 1 Assistant Professor, Jefferson University School of Population Health, 105 Walnut Street, Philadelphia, Pennsylvania, 19107, USA; 2 Research Assistant Professor, Georgetown University, 3700 Reservoir Road NW, Washington, DC, 20007 and O'Neill Institute for National and Global Health Law, Georgetown University Law Center, 600 New Jersey Avenue NW, Washington, DC, 20001, USA; 3 Georgetown University, 3700 Reservoir Road NW, Washington, DC, 20007, USA

## Abstract

**Background:**

School closure was employed as a non-pharmaceutical intervention against pandemic 2009 H1N1, particularly during the first wave. More than 700 schools in the United States were closed. However, closure decisions reflected significant variation in rationales, decision triggers, and authority for closure. This variability presents the opportunity for improved efficiency and decision-making.

**Methods:**

We identified media reports relating to school closure as a response to 2009 H1N1 by monitoring high-profile sources and searching Lexis-Nexis and Google news alerts, and reviewed reports for key themes. News stories were supplemented by observing conference calls and meetings with health department and school officials, and by discussions with decision-makers and community members.

**Results:**

There was significant variation in the stated goal of closure decision, including limiting community spread of the virus, protecting particularly vulnerable students, and responding to staff shortages or student absenteeism. Because the goal of closure is relevant to its timing, nature, and duration, unclear rationales for closure can challenge its effectiveness. There was also significant variation in the decision-making authority to close schools in different jurisdictions, which, in some instances, was reflected in open disagreement between school and public health officials. Finally, decision-makers did not appear to expect the level of scientific uncertainty encountered early in the pandemic, and they often expressed significant frustration over changing CDC guidance.

**Conclusions:**

The use of school closure as a public health response to epidemic disease can be improved by ensuring that officials clarify the goals of closure and tailor closure decisions to those goals. Additionally, authority to close schools should be clarified in advance, and decision-makers should expect to encounter uncertainty disease emergencies unfold and plan accordingly.

## Background

As concern about pandemic influenza in the United States grew in recent years, school closings increasingly were seen as a means of "social distancing" capable of slowing the spread of disease through the population [1]. Thus, when novel influenza H1N1 emerged suddenly in 2009, it was not surprising that over 726 K-12 schools in the United States closed, affecting 368,282 students [2,3]. However, school closure can cause lost instruction time and substantial economic losses for both families and educational facilities [4], as well as community upheaval. Because the decision to close schools is rife with challenges, clarifying and considering how to balance the multiple goals and objectives prior to a disease outbreak is an important component of public health emergency preparedness.

During the 2009 H1N1 outbreak in the United States there appeared to be extensive variation across the country in decisions made about school closures. As a result, decisions often appeared inconsistent, contributing to a sense that the government did not know how to respond, and perhaps was ineffective in meeting public health goals. In the systems improvement perspective, excess variation in health system structures and processes is a cause for concern, indicating possible inefficiencies and opportunities to improve the system [5,6]. Adopting this perspective, the goal of this paper is to identify the critical issues that arose during the Spring 2009 H1N1 outbreak, analyze sources of variation, and identify lessons learned to assist school districts and public health systems to improve their school closure decision-making process.

In particular, we describe three key issues that challenged decision-makers during the 2009 H1N1 outbreak: 1) clarifying goals, 2) clarifying authority and the decision-making process, and 3) dealing with uncertainty in preparing and issuing official guidance. Although we refer to a number of specific decisions that were made, our purpose is not to critique those decisions *per se *but rather to use them to identify problems that are likely to arise in future pandemics and other public health emergencies and learn from them to improve public health systems' capabilities. Similarly, we do not presume to say what school closing policies should be, but rather focus on the lessons learned from the 2009 experience to strengthen public health systems' response to future events.

## Methods

Many aspects of the 2009 H1N1 outbreak were well described in the media, on the Internet, and in scientific publications, so we have based this analysis primarily on monitoring key sources and Lexis-Nexis news and Internet searches. In particular, we identified media reports via Google alerts and Lexis-Nexis' database of all major newspapers in the United States using the search terms "swine flu," "H1N1," and "school closure" (as well as appropriate truncated and extended variations of these search terms). We included local and national media sources in the analysis, and we accepted overlapping stories in multiple sources in order to gain a full picture of events taking place. As appropriate, additional media reports were actively sought to better understand the context for particular localities' decisions. The purpose of the searches was not to systematically quantify each issue that was identified but, rather, to identify the scope of issues that arose and decisions that were made, so we stopped searching when new issues were no longer appearing. Our focus was on events during the Spring, but we consider some distinctions with the Fall in the discussion below.

At least two members of the research team read each media report and noted key issues that arose. In addition, we observed conference calls and meetings between health departments and school officials in the Boston and Washington, DC metropolitan areas, and we held informal interviews with decision-makers and community members as feasible. Many interviews were conducted *ad hoc *during flu clinics or meetings, as decision-makers did not have time for extensive formal interviews.

We organized the data collected from media reports and interviews into major themes, which we then vetted with public health researchers, practitioners, and education officials to ensure we captured the key issues and did not exclude any obvious concerns. While these methods cannot tell us the frequency with which specific issues arose, we are confident that we have identified and developed at least a basic understanding of the key public health issues.

## Results

### Timing and extent of school closings

School closures during the pandemic's first wave occurred in several rough phases. Initially, schools were closed on a somewhat *ad hoc *basis. After CDC issued preliminary guidance on April 26, schools with 2009 H1N1 cases generally closed for at least seven days. On May 1, CDC changed its guidance to suggest 14 days of closure, and most schools with cases closed with the intent to remain shuttered for 14 days. However, CDC guidance changed again on May 5 to say that closure was generally not necessary. After May 5, most schools did not close after a case was identified, with the notable exception of the New York City school system, which continued closing schools to protect particularly vulnerable children into June. A timeline of major school closure events is provided in Table 1.

**Table 1:** Timeline of school closures

April 23, 2009 - The first suspected 2009 H1N1 cases in a school in the United States are identified at St. Francis Preparatory School in New York City. Eight confirmed cases were reported on April 26 [7].
April 24, 2009 - All schools in Mexico City--serving about seven million students--are closed as a social distancing measure to slow the spread of 2009 H1N1 [8].

April 25, 2009 - Byron Steele High School in Cibolo, Texas, is the first school in the United States closed because of 2009 H1N1. The following day, all schools in Schertz-Cibolo-Universal City Independent School District are closed by the Texas Department of State Health Services [9].

April 26, 2009 - School officials close St. Francis Preparatory School to disinfect the school. The same day, CDC recommends closing schools for seven days when a case is identified [10].

May 1, 2009 - The U.S. Centers for Disease Control and Prevention recommends that schools close for up to 14 days if a case of 2009 H1N1 is identified [11].

May 5, 2009 - CDC changes its guidance to recommend that schools generally do not need to close and should, instead, keep ill children home for at least seven days [12].

June 2, 2009 - New York City closes its final school in response to 2009 H1N1. New York continued school closures well after CDC ceased recommending closure, with the justification that closure would protect particularly vulnerable students from within-school transmission [13].

August 7, 2009 - CDC recommends against school closures in most cases, instead recommending that ill children remain at home, as part of its comprehensive guidance for schools to use during the Fall semester [14].

### Rationale for school closure

Three different rationales for closing schools during a public health emergency emerged in the 2009 H1N1 outbreak: (1) limiting spread of the virus in the community, (2) protecting vulnerable children, and (3) reacting to staff shortages or children kept at home because of infection or parents' fears of infection.

In the United States as in Europe [15], the most common rationale for school closure was to limit spread of the H1N1 virus in the community. This is based on the idea that schools provide an ideal context for spread of infectious diseases because children are more susceptible to infection, less likely to adopt behavioral changes that reduce disease spread, and more likely to sustain person-to-person contact for lengthy periods. Closing schools, therefore, may limit spread among children as well as to their families and the general community, and can be an important component of a community's "social distancing" efforts [16]. Reducing community transmission might have the effect of reducing cumulative incidence in a community, but, more likely, would spread the epidemic curve in a community, slowing the accumulation of cases while alternative control measures are employed and reducing peak incidence to a level more manageable by the health care system [17]. Closing schools was an important component of Mexico's highly-regarded social distancing efforts in response to 2009 H1N1 [18]. U.S. school administrators and public health officials typically justified the costs of school closure on these grounds [19-21].

Evidence of the impact of school closure on the spread of influenza, however, is limited and mixed. Historical analyses and epidemiologic modeling studies for influenza and other respiratory diseases suggest that social distancing measures, especially if implemented early in an outbreak and sustained, can substantially reduce both the total number of cases and the peak attack rate [22-26]. On the other hand, a recent systematic review found only 19 studies with primary empirical data on the impact of school closure *per se*, many of which had significant methodological challenges [27]. Some mathematical models suggest that school closure has a limited impact on cumulative case counts, since children not attending school are free to transmit infection to their families and others in the community [28]. Other modeling studies suggest combining school closure with sequestering children in the home may curb pandemic spread and peak incidence [29]. Differences are largely attributable to assumptions underlying the models, reflecting uncertainties about epidemiology and behavior that may be better understood as more data become available. At minimum, though, it is clear that closure's effectiveness is reduced if students congregate in large number in other settings, and this proved to be a challenge in 2009 [30]. It also appears, based on modeling studies, that significant reductions in cumulative incidence may require extended periods of closure (with eight to sixteen weeks resulting in the greatest reductions in peak incidence) [31]. We did not identify any school that closed for this long, except in Hong Kong, where schools were closed and not reopened before summer break, with a reduction in cases occurring simultaneously [32]. This is the only epidemiological analysis of which we are aware to suggest that school closings actually had the effect of limiting spread of H1N1 in 2009.

Although advocated as a social distancing measure that imposes less societal costs than workplace closures or public disruptions, it became evident during the Spring 2009 H1N1 pandemic wave that school closures imposed substantial costs in some instances. Parents complained of the difficulty of finding child care or the financial costs associated with finding someone to take care of their children or staying home [33]. New York City officials originally based their decision to close schools one-at-a-time on the need to balance public health interests "with the child-care and educational needs of families" [34]. These officials noted, however, that closures were compromised as a control measure if "the kids don't go to school and instead go to the shopping mall or go to the park" [35] as library officials in Queens reported a large number of children congregating after their schools were closed [36].

Indeed, formal studies of students in Boston and Pittsburgh have documented this behavior. High school students in the Winsor School, a private girls' school in Boston that closed from May 20-26 following a sudden increase in absenteeism, reported the average number of days during this period on which they participated in the following activities: shopping 1.47 days, visiting a friend 2.21 days, using public transport 1.89 days, eating out 2.44 days, and outdoor activities 3.42 days [37]. During a one-week closure of an elementary school in southwestern Pennsylvania that closed during this period, 69% of students report having visited at least one location outside their home [38]. These findings are consistent with pre-pandemic findings about student behavior during closures for seasonal influenza [39].

As data emerged to suggest that school-aged children experienced a higher attack rate than other age groups and an unusually high rate of complications from the 2009 H1N1 virus [40], protecting children, especially those who may be particularly vulnerable to complications, became a second rationale for school closure during the 2009 outbreak. In New York City, where officials continued closing schools even after CDC stopped recommending it, Mayor Michael Bloomberg eventually clarified that school closure would not slow transmission and that closure "has absolutely nothing to do with the spread of the disease." Rather, the justification for closures shifted to preventing secondary cases among particularly vulnerable school contacts [41].

The third rationale was purely practical: staff shortages, whether due to actual illness or concerns about infection or children kept at home because parents feared they would become infected simply made it impossible to keep schools open. We did not observe this rationale in the Spring 2009 H1N1 outbreak, however closure due to very high absenteeism became common in the Fall resurgence. For example, several Connecticut schools closed in October because of high student absenteeism. "You can't teach with one- to two-thirds of the class absent," explained the superintendant of a district with closed schools [42]. In the last week of October 2009, about 350 schools were closed nationwide, a high proportion of which appeared to be in response to high absenteeism [43].

### Triggers for school closings

Modeling studies consistently show that, to be effective, school closure to limit community-wide transmission requires an early trigger, such as before 1-2% of the population is infected [44,45]. These results reflect the assumption that influenza transmission can occur before patients develop symptoms or in the presence of mild symptoms, so there can be a substantial number of infections before a sizable number are identified. Detection of a novel strain usually occurs after it is well-established in a locale, so the community-wide benefit of school closures is likely substantially diminished at that point [46]. Unless closure is very fast following the first identified cases or case detection is very efficient, it may be difficult for schools to close in time to be maximally effective. Indeed, in 2009 transmission was often not identified in a locale until clusters had already been detected in schools [47].

For epidemiological purposes H1N1 cases are defined (with ascending degrees of certainty) as "suspected," "probable," or "laboratory confirmed" and the initial 2009 H1N1 case definitions emphasized contact with other known cases [48]. In particular, a suspected case, based on symptoms, became a probable case if there were other cases in a child's school. As such, these definitions created a degree of circularity, since suspected cases in a school were sometime enough to make others into probable cases.

Most U.S. schools did not close in the Spring until the surfacing of a probable case or until higher than normal rates of influenza-like-illness were observed at the school [49,50]. In New York City, a private high school with students who had recently returned from Mexico became an early focal point for infections, and was closed shortly after cases were confirmed, but after at least eight children were ill. In Texas, however, the Fort Worth school district closed all 144 schools after one confirmed and three others suspected school-aged cases were reported [51]. In Montgomery County, Maryland, school officials closed a public high school after a single probable case was identified. At the same time, other Maryland schools with probable cases remained open after consulting local health officials, though this decision appears to have been driven by the belief that transmission within the schools had not occurred and secondary cases were unlikely [52].

We did not observe schools adopting formal thresholds for closure (for example a certain percentage of students identified as ill or absent), although these were used in Japan in Spring 2009 [53].

### Authority and decision-making process for school closures

Much of the variation in school closure decisions in 2009 was due to differences from one jurisdiction to another in whom the legal and practical authority for making decisions was vested. Depending on the jurisdiction, the legal authority to close schools in response to a public health threat may rest with school or health officials, at the state or local level. Additionally, in some jurisdictions, closure authority changes if an emergency has been declared, potentially in different ways depending on the form of the declared emergency [54]. Many states include school closure measures in their pandemic plans [55,56], but the plans are often vague about who has the authority to make the decision. It is not surprising, therefore, that there was substantial variation in decisions to close schools during April and May 2009 and conflict between authorities in some jurisdictions.

In Cibolo, Texas, the first American jurisdiction to close schools, the decision was made by state health officials [57]. In Fort Worth, Texas, on the other hand, local school officials made the decision to close schools district-wide, on the basis of advice from the local health department [58]. In Montgomery County, Maryland, shortly after health officials decided to close schools, the School Superintendent protested the decision in a memo to the county school board stating, "We do not believe that this is the right decision given the lack of compelling evidence for continued closure provided to us by state and county health officials" [59].

In New York City, public schools were closed by the city Schools Chancellor in consultation with the New York City Department of Health and Mental Hygiene [60]. In addition, the local Roman Catholic archdiocese independently closed some schools on suspicion of 2009 H1N1 cases [61]. It is unclear whether city officials had the authority to order private schools closed, but they did recommend closure [62].

In some instances, public health and school officials faced contradictory concerns. One frequent issue that arose dealt with laws mandating the number of instruction days schools must provide to receive state funding. In several states, schools that closed for public health purposes risked losing state education funding or incurring significant costs by extending the school year. Different states responded to this issue in different ways. Rhode Island law authorizes the state's education commissioner to issue waivers of the instruction days requirement for schools closed due to emergencies, and such waivers were granted to schools closed for 2009 H1N1 [63]. New York law authorized waivers for schools closed due to weather and other disruptions but not epidemics [64]. The state legislature responded by passing a law authorizing a waiver similar to that in Rhode Island [65]. Tennessee's legislature passed a similar bill [66]. Connecticut law authorized a waiver for "extreme circumstances" which the state education commissioner did not include closures for influenza, and the state legislature did not enact a proposed statutory exemption. Closed Connecticut schools had to reschedule classes to meet for 180 days [67]. State education officials in Alabama similarly indicated that any school closed for influenza would lose a portion of its state funding if it did not reschedule enough classes to meet the minimum number of instruction days [68].

### Official Guidance

Early in the Spring outbreak, state and local health departments followed CDC guidance that districts "consider adopting school dismissal." This guidance largely was influenced by early reports suggesting a high case-fatality rate from 2009 H1N1 and that youth may be at greater than average risk. CDC's initial guidance also suggested that schools with cases stay closed for 14 days [69], reflecting concerns about the risks of reopening while disease was still being transmitted. Such "guidance," often transmitted through state and local health departments, carries substantial weight in local decision-making. In part because state and local officials do not have the same epidemiological knowledge as CDC, guidance is often regarded as a recommendation.

CDC subsequently revised its guidance, announcing that schools closed under the prior guidance could reopen, but included the caveat that "decisions about school closure should be at the discretion of local authorities based on special circumstances and local considerations, including public concern and the impact of school absenteeism and staffing shortages" [70].

When additional data showed that the novel 2009 H1N1 was not especially severe, CDC changed its recommendation to keeping ill children at home [71]. Some schools, however, continued closing "to be on the safe side" [72].

Officials were often frustrated by frequent changes in the CDC guidance. For example, shortly after CDC increased its closure recommendation from 7 to 14 days, a Fort Worth, Texas, official stated, "The CDC is changing its plans and guidance on a daily basis" [73]. However, especially at the beginning of a disease outbreak, knowledge about disease severity, transmissibility, and the extent to which people with various underling conditions are at increased risk of complications is necessarily based on limited data. It should not be surprising for this information and the resulting guidance to be revised as more cases accumulate. Indeed in CDC's August 2009 school closure guidance, the agency notes that while it did not currently recommend closure, this could change if the disease's severity increased [74].

CDC's August 2009 school closure guidance suggests that local authorities make school closure decisions by balancing "the risks of keeping the students in school with the social disruption that school dismissal can cause" [75]. The guidance notes that "the potential benefits of preemptively dismissing students from school are often outweighed by negative consequences," but also that "school dismissals may be warranted, depending on the disease burden and other conditions." Recognizing that the severity may change, CDC has alternate guidance to be followed in the event of more serious disease [76].

## Discussion

The Institute of Medicine (IOM) describes a "public health system" as "a complex network of individuals and organizations that have the potential to play critical roles in creating the conditions for health" [77]. In the context of emergency preparedness, this includes "communities, health-care delivery systems, employers and business, the media, homeland security and public safety, academia, and the governmental public health infrastructure" [78]. The emergence of the novel H1N1 influenza in 2009 clearly showed unnecessary variation in the way that school closings were handled, suggesting problems in the public health ***system ***that must be addressed.

To address these problems, we have adopted a systems improvement (SI) approach [79,80], seeking to identify and reduce excess ***variability ***in processes and outcomes while preserving system differences in goals, context, and values that are critical to the specific environment [81]. Beyond this, excess variability is an indication suggests an opportunity for system improvement. This approach explicitly views a system's activities as defined processes, that is, chains of events that produce specific outcomes; focuses on changes that allow complex, intertwined systems of people and information to work more effectively; makes changes based on their effects on measurable outcomes; and encourages continuous improvement rather than onetime initiatives. SI efforts employ specific activities such as learning collaboratives, process analysis, and critical event or failure mode analysis. From this perspective, our analysis of school closings is an example of learning from a critical event about the nature and causes of variation in important aspects of the public health system's performance.

While CDC does not currently recommend large-scale school closure, questions about whether, when, and how to do so are likely to arise in the future, whether for 2009 H1N1 or some other pathogen. Because closure decisions require local public health concerns to be balanced with broader societal concerns, public health and school officials should consider the challenges inherent in closure and develop realistic plans to address them. Our analysis of the events of 2009 suggests three issues that require attention. First, as an outbreak develops, the goals of school closing should be clarified and specific measures adapted to the goals. Second, as part of planning and preparedness efforts, the legal and practical authority to close schools should be clarified. Finally, decision-makers should expect uncertainty and act accordingly.

### Clarifying goals and forms of school closing

As indicated above, three different and conflicting rationales for closing schools emerged in the 2009 H1N1 outbreak: (1) limiting spread of the virus in the community, (2) protecting vulnerable children, and (3) reacting to staff shortages or children kept at home because parents feared they would become infected. The rationale matters because it drives considerations such as whether to close schools at all, the nature and extent of closure, and the triggers for closure and re-opening. For example, because modeling data suggest that closure of one month or longer may be necessary to substantially reduce community transmission, closure for that purpose might have to be much longer (and entail more costs) than closure for other purposes [82]. Additionally, closure decisions require effective balancing of potential benefits and consequences, and this cannot be done without a clear idea of what benefits are desired.

Current CDC guidance recognizes the potential costs and difficulties of closure, calling for a careful balancing between potential benefits from closure and the potential "negative consequences, including students being left home alone, health workers missing shifts when they must stay home with their children, students missing meals, and interruption of students' education" [83]. While many parents and students will not find closure to be a significant hardship [84], officials considering closure must weigh not only the total amount of disruption but also the extent to which social costs will be disproportionately borne by certain segments of society, such as those who depend on school lunches to meet nutritional needs. Closure, if adopted, should be necessary to achieving goals which cannot effectively be achieved through lesser alternatives, such as requiring ill students to stay home or granting liberal absences. It is unclear to what extent school and health officials balanced hardships from closure against health protection, but such balancing played a role in CDC's decision to stop advising that schools close when it determined that A/H1N1 was less dangerous than initial reports suggested.

School closure can take a variety of forms [85]. One end of the spectrum is full closure: neither educational nor administrative functions continue and school personnel do not arrive for work. Alternatively, classes can be dismissed, but some or all administrative functions may continue in partial closures. Partial closures may also allow schools to continue providing meals or other social services during the period of class dismissal [86], and continue to hold social events such as proms, and academic gatherings such as SAT or ACT testing services. However, partial closure that allows students to congregate may defeat its purpose. During the Spring of 2009, it appears that the great majority of closures were full and conducted preemptively.

There are a variety of alternatives to closure that schools might consider. One of these is currently endorsed by CDC for most instances: requiring ill students to remain home in order to avoid infecting others [87]. Additionally, schools could reduce the likelihood that children with underlying conditions predisposing them to complications of 2009 H1N1 will be exposed by authorizing a liberal absence policy for those students. A school which considering whether to close to ameliorate public fears might relax attendance requirements for all students instead.

However, because public schools are funded based on student attendance levels, some schools may choose to close during times of high absenteeism in order to make classes up later in the year. Alternatively, they could attempt to overcome concerns about students missing class material by instituting online teaching, which at least one school in Maryland attempted [88]. Indeed, the U.S. Department of Education has suggested a variety of off-site continuity of instruction tools including the use of electronic media to teach children who are at home [89], although it is unclear how many teachers are prepared for distance teaching.

If the goal is to protect particularly vulnerable students, schools may wish to defer to parents' decisions about the costs and benefits of a vulnerable student missing school, and allow parents broader license to consult medical professionals and preemptively keep their children home. Other officials may prefer a blunter option--such as closure--on the grounds that parents may lack information to weigh risks and benefits. In addition, closing schools later in an outbreak may still serve to protect vulnerable children and staff from complications if within-school intensity of transmission is high.

### Clarifying legal authority for school closure

As described above, school closing decisions in 2009 were often inconsistent between neighboring jurisdictions and over time. This may have contributed to a sense that the government did not know how to respond, and perhaps was ineffective in meeting public health goals. Similar problems were seen during the 1918-19 pandemic when, as Stern and colleagues report, "ill-defined lines of authority among governmental branches contributed to the eruption of interagency conflict in U.S. cities ... [and] confusion about authority and jurisdiction helped lead to distrust in health officials and political leaders." The 2009 as well as the 1918-19 experiences suggests that, at the very least, officials must consider in advance who has the authority to close schools and identify what goals they wish to accomplish through a potential closure.

Inconsistencies of this sort can be particularly obvious in a region such as the Washington DC metropolitan area, which includes two states as well as the District of Columbia, plus numerous local jurisdictions, but one media market. In such settings, and even in isolated jurisdictions, it seems important to be sure that reasons for differences in decisions be clearly communicated to the public.

Regardless of who has the formal authority to close schools in a jurisdiction, the 2009 experience shows that it is helpful to solicit input from a range of stakeholders such as local and state health and school officials as well as students, their parents, and school staff. Broader inclusion of stakeholders both improves the likelihood that decisions are made with full information and promote consideration of benefits and costs that will accrue to different affected groups. Coordination of this sort also helps to ensure the credibility of the message [90]. The New York school closures described above, for instance, conveyed a sense of a unified city government decision, announced jointly by the top-ranking school and health officials, and press announcements also frequently included Mayor Michael Bloomberg.

### Expect uncertainty

Decisions about whether and how to close schools logically depend on epidemiological information about who is likely to be infected, the severity of illness in those infected, and periods of infectiousness. Infectious disease outbreaks, however, are often characterized by scientific uncertainty [91], and the 2009 H1N1 outbreak was no different. Changing and variable case definitions led to uncertain understanding of the epidemiological risks, which in turn led to frequent changes in official guidance. This uncertainty made all of the decisions regarding school closing more difficult. In particular, information emerged during the Spring to suggest that, 2009 H1N1 infection less severe than most pandemic planning assumptions, and children were more likely to be infected and suffer severe consequences. Each of these factors influenced decisions about whether and how to close schools.

If the 2009 H1N1 pandemic is any guide to the future, public health and other officials should expect and plan for uncertainty about the facts and frequent changes in official guidance that is based on a constantly evolving epidemiologic knowledge base. This begins with acknowledging the uncertainty and requires flexibility in policies and procedures such as consideration of variations of school closing and alternatives as discussed above. During the 2009 H1N1 pandemic, for instance, the goal of the acting CDC Director Richard Bessor was to "tell everything we knew, everything we didn't know, and what we were doing to get the answers" [92].

In particular, because H1N1 has and will likely continue to affect communities differentially; both socially and epidemiologically, monitoring the situation at the local level will play an important role in closure decisions. Communities will experience varying levels of transmission, and some populations have more people who are highly susceptible to complications than others. Wealthier communities may be able to keep schools closed as parents have childcare alternatives, while communities with more disadvantaged populations may depend on schools for a variety of resources such as childcare and school lunches. The 2009 experience suggests that local school and health officials must monitor the situation in their communities and work together closely to integrate local information with state and federal guidance.

Planning for uncertainty includes development of systems to track the epidemic and its consequences as well as evaluate the impact of control efforts, that is, to provide "situational awareness." It also requires a degree of humility in presenting decisions to both senior policy makers and the public, clearly stating the basis on which decisions were made and noting the likelihood that they can, or are likely to, change.

## Conclusion

A careful analysis of the 2009 H1N1 outbreak finds extensive variation across the United States in expressed rationales, decision triggers, and decision-making authority for school closures. This led to decisions which were often inconsistent from one locale to another and over time, contributing to a sense that the government was unsure how to respond and perhaps ineffective. From a systems improvement perspective, however, such excess variation in is a cause for concern, indicating possible inefficiencies and opportunities to improve the system.

Because school closure decisions require local public health concerns to be balanced with broader societal concerns, analysis of the 2009 H1N1 pandemic suggests three issues public health and school officials should consider in planning for and making school closure decisions in the future. First, the goal of school closing should be made clear and specific measures should be tailored to the goal and modified over time as evolving knowledge requires. Second, legal and practical authority to close schools should be clarified in advance, as part of planning and preparedness efforts. Finally, decision-makers should expect uncertainty and maintain situational awareness, be flexible in policies and procedures, and act with humility.

## Competing interests

The authors declare that they have no competing interests.

## Authors' contributions

JK and TK searched the literature to identify information for this analysis. All authors participated in designing the study, analyzing and interpreting the data, drawing policy conclusions, and drafting the manuscript. All authors read and approved the final manuscript.

## Pre-publication history

The pre-publication history for this paper can be accessed here:

http://www.biomedcentral.com/1471-2458/11/73/prepub
